# Maternal safety outcomes following bilateral hypogastric artery ligation in placenta accreta spectrum and severe obstetric hemorrhage: a retrospective tertiary-center cohort

**DOI:** 10.3389/fmed.2026.1962336

**Published:** 2026-10-21

**Authors:** Petra Hanulikova, Ladislav Krofta, Matus Halaj, Lubomir Haslik, Daniela Vankova, Hooman Soleymani majd

**Affiliations:** 1 Department of Obstetrics and Gynecology, Institute for the Care of Mother and Child, Third Faculty of Medicine, Charles University, Prague, Czechia; 2 Department of Gynecological Oncology, Oxford University Hospitals NHS Foundation Trust, Churchill Hospital, Oxford, United Kingdom; 3 Nuffield Department of Women’s & Reproductive Health, University of Oxford, Oxford, United Kingdom

**Keywords:** hypogastric artery ligation, internal iliac artery ligation, maternal morbidity, obstetric hemorrhage, placenta accreta spectrum, surgical safety

## Abstract

**Objective:**

The hemostatic efficacy of bilateral hypogastric artery ligation (HAL) remains controversial, whereas its procedure-related maternal safety has been insufficiently investigated. This study evaluated procedure-related maternal morbidity and long-term patient-reported outcomes following prophylactic or therapeutic HAL.

**Methods:**

This retrospective cohort study included 137 women with complete outcome data who underwent bilateral hypogastric artery ligation (HAL) at a tertiary referral center between 2005 and 2023. Women were stratified according to PAS status (PAS group, *n* = 45; non-PAS group, *n* = 92); all PAS cases had been suspected antenatally. The primary outcome was early non-transfusion major maternal morbidity, defined as disseminated intravascular coagulation, urinary tract injury, or reoperation within 7 days after delivery. Secondary analyses evaluated PAS severity (FIGO grades 1–2 vs. 3a/b), long-term patient-reported outcomes, and the independent association between PAS status and major maternal morbidity.

**Results:**

Women with PAS delivered earlier (35 vs. 37 weeks, *p* < 0.001) and underwent hysterectomy more frequently (84.4% vs. 34.8%, *p* < 0.001). Despite substantially greater surgical complexity, early procedure-related maternal morbidity was comparable between groups (24.4% vs. 23.9%, *p* = 1.000). Urinary bladder injury occurred more frequently in the PAS group (17.8% vs. 3.3%, *p* = 0.006), whereas rates of disseminated intravascular coagulation and reoperation were similar. Within the PAS group, FIGO grade 3a/b was associated with higher rates of overall complications and massive transfusion. Multivariable analysis demonstrated that PAS status was not independently associated with early major maternal morbidity after adjustment for gestational age, previous uterine surgery, maternal age, BMI, and parity. There were no ischemic complications in long-term patient-reported outcomes (*n* = 63).

**Conclusions:**

Our findings provide clinically relevant evidence on procedure-related maternal outcomes following HAL when performed within an experienced multidisciplinary tertiary-center setting. Although PAS was associated with substantially greater surgical complexity, early procedure-related maternal morbidity remained comparable between groups, and no severe long-term ischemic complications were reported in women who completed the structured follow-up questionnaire.

## 1 Introduction

The rising global incidence of placenta accreta spectrum (PAS) represents a challenge in contemporary obstetrics, primarily driven by the increasing rates of cesarean deliveries (1). PAS is associated with life-threatening peripartum hemorrhage, frequently necessitating multidisciplinary surgical interventions to reduce maternal morbidity and mortality (2). While prenatal diagnosis has improved, managing the profound pelvic hypervascularity remains a complex task, often requiring advanced devascularization techniques (3).

Bilateral hypogastric artery ligation (HAL), also referred to as internal iliac artery ligation, has been used for more than a century in the management of severe pelvic hemorrhage. Although originally introduced as a life-saving hemostatic procedure, its contemporary role has evolved into a standardized, multidisciplinary surgical approach for complex obstetric hemorrhage, particularly placenta accreta spectrum (PAS) (4, 5).

Despite its long history, the role of HAL in PAS remains controversial. Experimental physiological studies demonstrated that extensive pelvic collateral circulation limits the reduction in arterial blood flow following internal iliac artery occlusion (5, 6). Accordingly, international consensus statements and clinical guidelines do not recommend routine use of this technique solely to reduce blood loss, as randomized trials and systematic reviews have generally failed to demonstrate consistent reductions in hemorrhage or transfusion requirements (7–14).

Nevertheless, surgical HAL continues to be incorporated into standardized multidisciplinary PAS protocols in many high-volume referral centers (15). This apparent discrepancy reflects the fact that HAL is often used not as an isolated hemostatic intervention but as one component of a broader surgical strategy aimed at facilitating pelvic devascularization and complex retroperitoneal dissection.

Importantly, most available studies have focused on the hemostatic efficacy of internal iliac artery occlusion. Far less attention has been paid to an equally important question—its procedural safety**.**

Furthermore, comparisons between prophylactic and therapeutic HAL are inherently susceptible to confounding by indication because women undergoing rescue ligation usually present with substantially more severe hemorrhage than those treated prophylactically (16).

Therefore, the objective of the present study was not to reassess the hemostatic efficacy of HAL but to evaluate its safety profile in contemporary obstetric practice. Specifically, we compared early non-transfusion maternal morbidity between women undergoing prophylactic HAL during planned PAS surgery and those requiring therapeutic HAL for severe obstetric hemorrhage, while also assessing long-term patient-reported outcomes using a structured questionnaire.

## 2 Methods

### 2.1 Study design and population

We conducted a retrospective cohort study at a tertiary referral center specializing in high-risk obstetrics and placenta accreta spectrum (PAS). The study population comprised 141 consecutive women who underwent bilateral hypogastric (internal iliac) artery ligation (HAL) as part of planned PAS surgery or for the management of severe obstetric hemorrhage between January 2005 and December 2023. After exclusion of four women with incomplete outcome data, 137 women were included in the primary outcome analysis (Figure 1).

**Figure 1:**
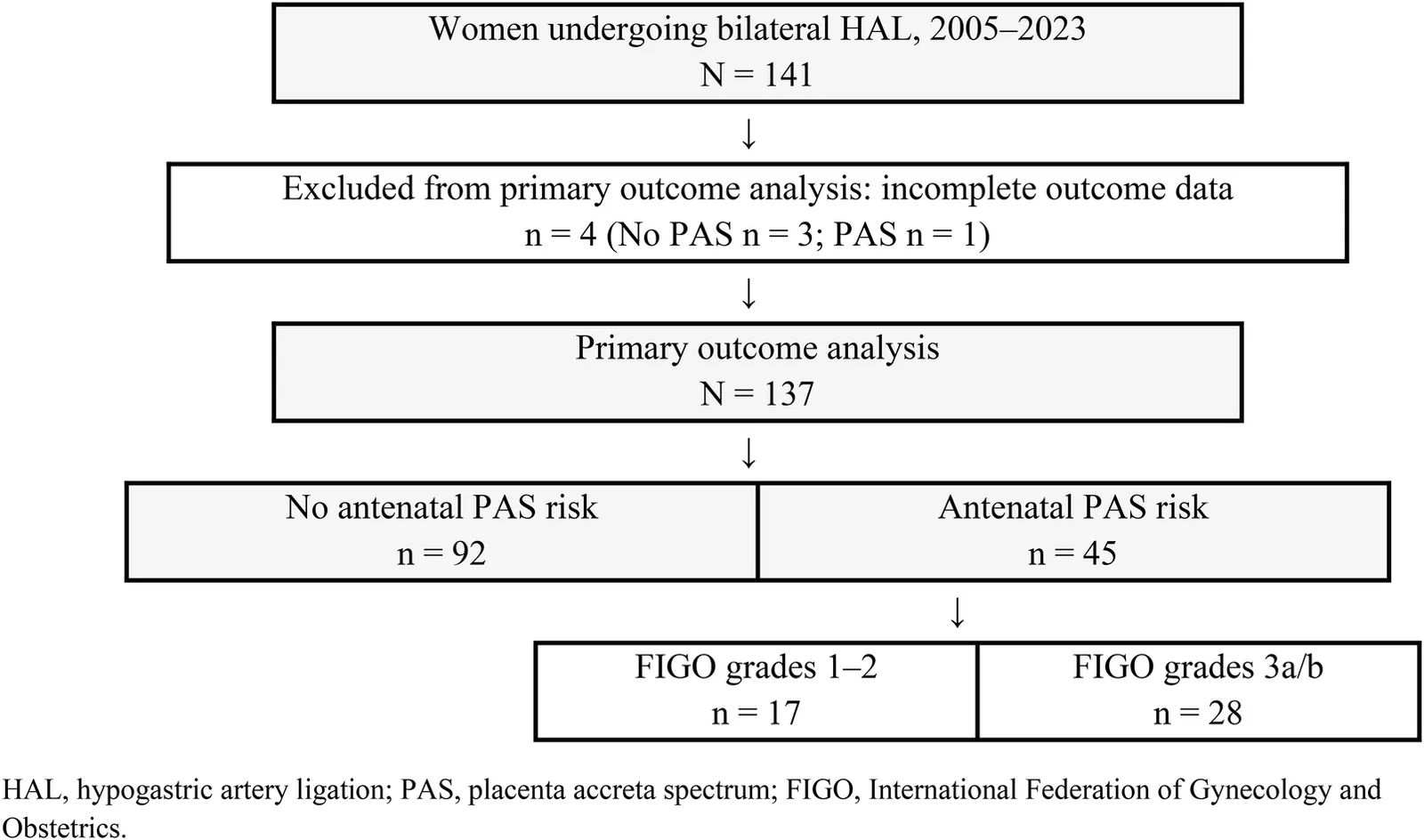
Flowchart.

The study was approved by the Ethics Committee of the Institute for the Care of Mother and Child (approval No. 3/25/9/2020).

### 2.2 Patient stratification and PAS confirmation

Patients were stratified into two groups according to PAS status:

PAS Group (*n* = 45): Women with confirmed PAS. All PAS cases had been suspected antenatally. HAL was typically performed as a prophylactic first-step procedure. PAS was confirmed postpartum using histopathological examination (uterus with placenta when hysterectomy was performed or placental specimens) and the FIGO clinical classification (2). Cases managed before publication of the FIGO 2019 classification were retrospectively graded according to the FIGO PAS classification by two senior specialists using operative records and definitive histopathological reports.

Non-PAS Group (*n* = 92): Women without PAS. In this group, HAL was predominantly performed therapeutically as a rescue procedure for acute obstetric hemorrhage, including uterine atony, genital tract trauma following vaginal delivery, and hemoperitoneum. Prophylactic HAL was performed in 13 women, all with placenta previa.

### 2.3 Surgical technique

The ligation of the internal iliac arteries was performed via a transperitoneal approach. Following entry into the retroperitoneum and the identification of the common iliac artery bifurcation, the ureter was carefully isolated and retracted medially to prevent inadvertent injury. The internal iliac artery was carefully dissected approximately 2–3 cm distal to the bifurcation of the common iliac artery. Following adequate mobilization and creation of a retroarterial passage, the artery was ligated using non-absorbable silk sutures.

Women with antenatally suspected PAS were managed according to a standardized multidisciplinary surgical protocol. In the majority of these cases, placental invasion involved the anterior lower uterine segment, typically associated with placenta previa and a previous cesarean scar. The surgical strategy prioritized early pelvic devascularization through HAL, followed by meticulous bladder dissection and clear identification of the ureters. The systematic development of the paravesical and pararectal spaces was essential to ensure adequate surgical exposure and safety before proceeding to definitive surgical management.

A comprehensive description of the surgical technique and specific anatomical considerations is available in the Supplementary Materials.

### 2.4 Outcome Measures

The primary outcome was early non-transfusion major maternal morbidity occurring within 7 days after the index delivery. This composite outcome included:

- disseminated intravascular coagulation (DIC),
- urinary tract injury (bladder or ureter),
- early reoperation.

Transfusion requirements were intentionally excluded from the primary composite outcome because they primarily reflect the severity of hemorrhage rather than the safety of the surgical procedure itself. This methodological approach was chosen to minimize confounding by indication and to specifically evaluate procedure-related maternal morbidity. Since severe hemorrhage was the dominant indication for therapeutic HAL in the non-PAS group, including transfusion as a morbidity marker would have disproportionately weighted the results against the rescue cohort and complicated interpretation of the comparison.

### 2.5 Definitions of complications

Any early maternal complication was defined as the occurrence of any of the following within 7 days after delivery: transfusion, DIC, urinary tract injury, or early reoperation.

Disseminated intravascular coagulation was classified retrospectively according to the 2025 International Society on Thrombosis and Haemostasis (ISTH) scoring system (17). The laboratory parameters required for retrospective DIC assessment were available for all patients.

Early reoperation was defined as any unplanned surgical intervention performed within 7 days after the index delivery because of hemorrhagic, infectious, or other surgery-related complications. This definition was based on previously published composite maternal morbidity outcomes in placenta accreta spectrum research (18) and is consistent with the Clavien–Dindo classification of postoperative complications (19).

Long-term functional outcomes were assessed using a standardized questionnaire administered during routine follow-up or structured telephone interviews. The questionnaire focused on potential late complications related to HAL, including dyspareunia, urinary incontinence, ischemic pelvic pain, and soft tissue necrosis. Of the original cohort of 141 women, 75 were approached for long-term follow-up, of whom 63 completed the questionnaire and were included in the long-term patient-reported outcome analysis

### 2.6 Statistical analysis

Continuous variables were expressed as median and interquartile range (IQR). Differences between the PAS and non-PAS groups and between FIGO grade up to 2 and FIGO grade 3 groups were analyzed using the Mann–Whitney U test or Fisher's exact test. Multivariable logistic regression was performed to evaluate whether PAS status was independently associated with early non-transfusion major maternal morbidity, adjusting for maternal age, BMI, parity, gestational age at delivery, and a history of previous uterine surgery. All statistical computations were performed in IBM SPSS Statistics 30.

## 3 Results

### 3.1 Baseline characteristics

The study included 137 women, categorized as non-PAS group (*n* = 92; 67.2%) or PAS group (*n* = 45; 32.8%). Demographic and clinical characteristics are detailed in Table 1. No significant difference was found in median maternal age (33 vs. 34 years, *p* = 0.409). The PAS group was characterized by significantly lower gestational age at delivery (35 vs. 37 weeks, *p* < 0.001) and a prevalence of placenta previa and previous uterine surgery (100% vs. 22.8% and 100% vs. 21.7%, respectively; both *p* < 0.001). Consequently, the PAS group required cesarean section in all cases and underwent hysterectomy significantly more often than the non—PAS group (84.4% vs. 34.8%, *p* < 0.001). HAL was performed prophylactically in all patients from the PAS group, whereas it was predominantly a therapeutic measure (85.9%) in the non–PAS group.

**Table 1:** Baseline characteristics according to PAS status.

Parameter	Non- PAS group	PAS group	*p*
	N-92 (67.2%)	N-45 (32.8%)	
Median mother age at birth (years)	33 (31–37)	34 (32–36)	0.409*
Gestation week at delivery	37 (33–39)	35 (33–36)	< 0.001*
Placenta praevia			
No	71 (77.2%)	0 (0.0%)	**< 0.001** ^+^
Yes	21 (22.8%)	45 (100.0%)	
History of previous uterine surgery			
No	72 (78.3%)	0 (0.0%)	**< 0.001** ^+^
Yes	20 (21.7%)	45 (100.0%)	
Cesarean section			
No	24 (26.1%)	0 (0.0%)	**<0.001** ^+^
Yes	68 (73.9%)	45 (100.0%)	
Hysterectomy			
No	60 (65.2%)	7 (15.6%)	**<0.001** ^+^
Yes	32 (34.8%)	38 (84.4%)	
HAL indication			
Therapeutic	79 (85.9%)	0 (0.0%)	**<0.001** ^+^
Prophylactic	13 (14.1%)	45 (100.0%)	

Presents baseline characteristics according to PAS status. Numerical variables are reported as median with lower and upper quartiles. Categorical variables are reported as frequency (n) and column (%). The bold-emphasized variables show statistically significant (at a 5% significance level) differences in proportion between groups.

Statistics: *Mann–Whitney U test, +Fisher's exact test.

### 3.2 Early maternal outcomes

The primary outcome—early non-transfusion major maternal morbidity—demonstrated no statistically significant difference between the groups, occurring in 11/45 (24.4%) of the PAS group and 22/92 (23.9%) of the non-PAS group (*p* = 1.000). Specific outcomes are summarized in Table 2. While the incidence of DIC (13.3% vs. 17.4%, *p* = 0.627) and reoperation within seven days (2.2% vs. 12.0%, *p* = 0.103) did not differ significantly, urinary tract injury was significantly more frequent in the PAS group (17.8% vs. 3.3%, *p* = 0.006); all injuries involved the urinary bladder, with no ureteral injuries observed. Median blood loss (1,700 mL vs. 1,500 mL, *p* = 0.169) and median operation time (122.5 vs. 120 min, *p* = 0.121) were comparable. Massive transfusion (≥4 units RBC) was required in approximately 40% of patients in both groups (*p* = 0.717).

**Table 2:** Early maternal outcomes according to PAS status.

Parameter	Non-PAS group	PAS group	*p*
	N-92 (67.2%)	N-45 (32.8%)	
Primary outcome: non-transfusion major morbidity			
No	70 (76.1%)	34 (75.6%)	1.000^+^
Yes	22 (23.9%)	11 (24.4%)	
DIC			
No	76 (82.6%)	39 (86.7%)	0.627^+^
Yes	16 (17.4%)	6 (13.3%)	
Urinary tract injury (bladder only)			
No	89 (96.7%)	37 (82.2%)	**0**.**006**^+^
Yes	3 (3.3%)	8 (17.8%)	
Reoperation ≤7 days			
No	81 (88.0%)	44 (97.8%)	0.103^+^
Yes	11 (12.0%)	1 (2.2%)	
Transfusion ≥4 units RBC			
No	52 (56.5%)	27 (60.0%)	0.717^+^
Yes	40 (43.5%)	18 (40.0%)	
Blood loss (mL)	1,500 (800–2,075)	1,700 (1,000–2,500)	0.169*
Operation time (min)	120.0 (90.0–145.0)	122.5 (106.3–171.3)	0.121*

Presents early maternal outcomes according to PAS status. Numerical variables are reported as median with lower and upper quartiles. Categorical variables are reported as frequency (n) and column (%). The bold-emphasized variables show statistically significant (at a 5% significance level) differences in proportion between groups.

Statistics: *Mann–Whitney U test, +Fisher's exact test.

No bowel injuries, major vascular injuries, or other catastrophic intraoperative complications occurred in this cohort. Postoperative intensive care admission was excluded from the outcome analysis because it is routine institutional practice for all women undergoing major obstetric surgery at this center.

### 3.3 Impact of PAS severity (FIGO grade)

Analysis of the PAS group based on FIGO classification revealed that severity significantly influenced surgical outcomes (Table 3). Women with FIGO grade 3a/b (*n* = 28) experienced a significantly higher rate of any early complication compared to those with FIGO grade 1-2 (*n* = 17; 57.1% vs. 23.5%, *p* = 0.035). Furthermore, massive transfusion was more frequent in the FIGO 3a/b group (53.6% vs. 17.6%, *p* = 0.028). All patients with FIGO 3a/b required a hysterectomy, while uterine preservation was achieved in 41.2% of FIGO 1-2 cases (*p* < 0.001). Within the FIGO 3a/b subgroup (*n* = 28), four cases (14.3%) were specifically identified as FIGO Grade 3b (clinical evidence of bladder invasion). Notably, all four Grade 3b cases required intraoperative bladder repair, significantly contributing to the overall urological morbidity observed in the PAS cohort.

**Table 3:** Maternal outcomes according to PAS severity.

Complication	FIGO 1-2	FIGO 3 a/b	*p*
	N-17 (37.8%)	N-28 (62.2%)	
Any early maternal complication, *n* (%)			
No	13 (76.5%)	12 (42.9%)	**0**.035*
Yes	4 (23.5%)	16 (57.1%)	
Primary outcome: non-transfusion major morbidity			
No	15 (88.2%)	19 (67.9%)	0.165*
Yes	2 (11.8%)	9 (32.1%)	
DIC			
No	16 (94.1%)	23 (82.1%)	0.385*
Yes	1 (5.9%)	5 (17.9%)	
Urinary tract injury (bladder only)			
No	15 (88.2%)	22 (78.6%)	0.464*
Yes	2 (11.8%)	6 (21.4%)	
Reoperation ≤7 days			
No	16 (94.1%)	28 (100.0%)	0.378*
Yes	1 (5.9%)	0 (0.0%)	
Transfusion ≥4 units RBC			
No	14 (82.4%)	13 (46.4%)	**0**.028*
Yes	3 (17.6%)	15 (53.6%)	
Hysterectomy			
No	7 (41.2%)	0 (0.0%)	< 0.001*
Yes	10 (58.8%)	28 (100.0%)	

Presents complications according to FIGO grade. Variables are reported as frequency *n* and column %. The bold-emphasized variables show statistically significant (at a 5% significance level) differences in proportion between groups.

Statistics: *Fisher exact test.

Despite these differences in overall complexity, the primary composite outcome of non-transfusion major morbidity did not reach statistical significance between the severity grades (32.1% vs. 11.8%, *p* = 0.165).

### 3.4 Multivariable analysis

Binary logistic regression was performed to assess whether PAS status was independently associated with the primary outcome after adjustment for relevant baseline maternal and pregnancy characteristics (Table 4). The final model included maternal age, BMI, parity, gestational age at delivery, and previous uterine surgery. Placenta previa and HAL indication were also considered but were not retained because of substantial overlap with PAS status and numerical instability resulting from zero cells in some predictor–outcome combinations. Potential multicollinearity was assessed using adjusted generalized variance inflation factors (GVIF); all values were below √5 (approximately 2.24), indicating no evidence of problematic multicollinearity. PAS status was not independently associated with early non-transfusion major maternal morbidity (adjusted OR 0.795, 95% CI 0.234–2.704; *p* = 0.714). The model showed limited discriminatory performance (AUC = 0.610); however, it was intended to estimate the adjusted association with PAS status rather than to serve as a predictive model.

**Table 4:** Multivariable logistic regression for primary outcome.

Effect	OR (95% CI)	*p*	adj. GVIF
PAS status	0.795 (0.234–2.704)	0.714	1.528
Age	1.066 (0.971–1.169)	0.178	1.031
BMI	0.993 (0.890–1.107)	0.894	1.033
Para 1	1.537 (0.517–4.566)	0.439	1.127
Para 2 or more	1.333 (0.349–5.093)	0.675	
Gestation week at delivery	1.024 (0.925–1.133)	0.645	1.059
Previous uterine surgery	1.167 (0.352–3.865)	0.801	1.587

Presents adjusted odds ratios for the primary outcome (AUC 0.610).

### 3.5 Long-term patient-reported outcomes

Long-term patient-reported outcomes were available for 63 of 141 women (44.7%) who completed the standardized questionnaire. Baseline characteristics did not differ significantly between women approached for long-term follow-up (*n* = 75) and those not approached (*n* = 62) (Supplementary Table S1). The questionnaire included symptoms potentially related to pelvic ischemia and pelvic floor dysfunction, including dyspareunia, urinary incontinence, ischemic pelvic pain, and soft tissue complications (Table 5). The frequency of reported outcomes did not differ significantly between the PAS and non-PAS groups. Dyspareunia was reported by 22.2% of women with PAS and 24.4% of those without PAS (*p* = 1.000), while urinary incontinence occurred in 5.6% and 22.2%, respectively (*p* = 0.155). Importantly, no woman reported ischemic pelvic pain or soft tissue necrosis, providing no evidence of severe long-term ischemic complications attributable to HAL.

**Table 5:** Long-term patient-reported outcomes following HAL.

Long-term outcome	Non-PAS group (*n* = 45)	PAS group (*n* = 18)	*p*
Any specific complication	16 (35.6%)	4 (22.2%)	0.379
Urinary incontinence	10 (22.2%)	1 (5.6%)	0.155
Dyspareunia	11 (24.4%)	4 (22.2%)	1.000
Ischemic pain	0	0	–
Soft tissue necrosis	0	0	–

Long-term outcomes were available for 63/141 women (44.7%). Individual symptoms cannot be attributed specifically to HAL without baseline data or a non-HAL comparator.

Statistics: Fisher exact test.

## 4 Discussion

### 4.1 Principal findings

In this real-world tertiary-center cohort, bilateral hypogastric artery ligation (HAL) was associated with comparable rates of early non-transfusion major maternal morbidity among women in PAS and non—PAS groups despite substantially greater operative complexity in the PAS cohort. Women with PAS underwent hysterectomy considerably more frequently, and more than 60% had invasive disease (FIGO grades 3a/b). Nevertheless, the observed rate of the composite primary outcome did not differ significantly between groups. As expected, urinary tract injury occurred significantly more often in women with PAS, with all injuries involving the urinary bladder, reflecting the anatomical extent of placental invasion rather than the ligation procedure itself. After adjustment for relevant baseline maternal and pregnancy characteristics, PAS status was not independently associated with early major maternal morbidity. Importantly, no severe long-term ischemic complications attributable to HAL were identified.

Taken together, these findings suggest that the clinical value of HAL should not be judged solely according to its hemostatic efficacy. Rather, our findings are reassuring regarding procedure-related maternal morbidity when HAL is incorporated into a standardized multidisciplinary surgical protocol in an experienced tertiary referral center. Because all women in this cohort underwent HAL and the groups differed in clinical indication, these findings characterize maternal outcomes following HAL but do not establish its comparative safety or hemostatic efficacy.

### 4.2 The methodological rationale: addressing confounding by indication

A primary challenge in evaluating HAL efficacy lies in the inherent risk of confounding by indication. El-Sherbiny et al. (16) recently emphasized that many studies fail to distinguish between complications arising from the underlying pathology and those directly attributable to the surgical intervention. By adopting a non-transfusion morbidity model, this analysis reduces the influence of transfusion-related confounding but does not isolate the effect of HAL. Although the primary purpose of HAL is pelvic devascularization aimed at facilitating hemorrhage control, its clinical role should not be judged solely according to reductions in blood loss. This concept is further supported by the recently developed Soleymani and Collins Obstetric Morbidity Score (SaCOMS), which was specifically designed to quantify maternal morbidity associated with complex obstetric surgery rather than relying solely on hemorrhage-related outcomes (20). Although our study did not apply the SaCOMS score retrospectively, the rationale underlying our composite non-transfusion endpoint is conceptually aligned with this contemporary shift toward procedure-related assessment of maternal surgical morbidity.

### 4.3 PAS complexity, FIGO 3b, and urological outcomes

The significantly higher rate of urinary tract injuries in the PAS group (17.8% vs. 3.3%, *p* = 0.006) reflects the advanced stage of placental invasion. Within our cohort, four cases were specifically identified as FIGO Grade 3b (bladder invasion). As noted by Lucidi et al. (21), urological morbidity is often an unavoidable consequence of Grade 3 PAS. However, by utilizing the systematic development of the paravesical and pararectal spaces—as advocated in the Soleymani–Alazzam–Collins technique (15) and further refined in recent stepwise devascularization protocols (22)—we achieved safe medial retraction of the ureters. This anatomical approach ensures that while bladder injury may occur due to the pathology, catastrophic ureteral injuries are avoided.

### 4.4 The HAL controversy: hemostatic efficacy versus procedural safety

The role of internal iliac artery occlusion in PAS remains controversial. Based on physiological studies demonstrating extensive pelvic collateral circulation, Nieto-Calvache et al. questioned the routine use of internal iliac artery occlusion as a primary hemostatic strategy, citing randomized trials and systematic reviews that failed to demonstrate a clinically meaningful reduction in blood loss or transfusion requirements with prophylactic balloon occlusion or surgical ligation (14).

Importantly, our findings should not be interpreted as contradicting these observations. Rather, our study addresses a different clinical question. Whereas previous studies primarily evaluated the hemostatic efficacy of internal iliac artery occlusion, our primary objective was to assess the procedural safety of surgical HAL. By intentionally excluding transfusion-related outcomes from the primary composite endpoint, we sought to minimize indication-based confounding and specifically evaluate procedure-related maternal morbidity.

Although our study was not designed to evaluate the hemostatic efficacy of HAL, this should not be interpreted as evidence against its potential surgical role within a standardized multidisciplinary PAS protocol. Physiological studies indicate that extensive collateral pelvic circulation limits sustained reductions in pelvic blood flow following internal iliac artery occlusion. Nevertheless, prophylactic HAL performed before major pelvic dissection may transiently reduce pelvic arterial pulse pressure, thereby facilitating retroperitoneal dissection and vascular control during the most demanding stages of PAS surgery before collateral circulation becomes fully established. This concept, advocated by Soleymani Majd and Collins (15), may explain why HAL continues to be incorporated into standardized multidisciplinary PAS protocols in experienced tertiary referral centers despite the absence of convincing evidence for reduced blood loss or transfusion requirements.

Furthermore, compared with interventional radiology, Papillon-Smith et al. (23) reported comparable maternal outcomes with surgical ligation, without the need for hybrid operating theatres. This may be particularly relevant in emergency situations where prenatal diagnosis is uncertain and vascular control strategies requiring preoperative radiological access cannot be implemented. Such scenarios remain common even in tertiary referral centers, as demonstrated in our previous study on emergency deliveries in suspected PAS (24).

### 4.5 Safety profile and long-term functional impact

Long-term patient-reported outcomes were obtained from 63 women who completed the structured questionnaire. The absence of catastrophic ischemic complications is reassuring, although the limited number of respondents means that rare adverse events cannot be excluded. Patient-reported outcomes, including dyspareunia and urinary incontinence, occurred with similar frequency in women with and without PAS, suggesting that these symptoms may be related to pregnancy, childbirth, and complex pelvic surgery rather than HAL itself. However, these findings should be interpreted with caution because baseline functional status was unavailable and no comparator group without HAL was included. As previously highlighted by Sentilhes et al. (19), attributing long-term pelvic floor symptoms specifically to HAL is difficult given the high proportion of women undergoing cesarean hysterectomy and the substantial physiological impact of severe obstetric hemorrhage and complex pelvic surgery.

### 4.6 Strengths and limitations

The primary strength of this study lies in the size of the cohort (*n* = 141), which represents a substantial sample for a specialized surgical intervention. The data reflect real-world clinical practice in a high-volume tertiary center, providing consistency in surgical technique and perioperative management. Methodological rigor was further enhanced by using the FIGO classification for PAS confirmation and severity grading, enabling detailed comparisons of outcomes across different degrees of placental invasion. Furthermore, the inclusion of long-term patient-reported outcomes addresses a significant gap in current literature, which predominantly focuses on immediate perioperative survival rather than functional recovery.

Several limitations must be acknowledged. The retrospective design carries inherent risks of selection and information bias. Because all women in this cohort underwent HAL, the absence of a comparator group managed without HAL precludes conclusions regarding the comparative safety or hemostatic efficacy of the procedure. Despite multivariable adjustment, residual confounding cannot be excluded because of the retrospective design and substantial differences in clinical indication and baseline characteristics between the PAS and non-PAS groups. Moreover, the specialized expertise of a high-volume tertiary referral center may limit the generalizability of these findings to lower-volume or less experienced settings. Additionally, the primary outcome excluded transfusion requirements to avoid confounding by the baseline clinical condition (hemorrhage as the indication for HAL), which may limit direct comparability with studies using broader composite scores. Finally, long-term patient-reported outcomes were obtained from only a subset of the cohort (*n* = 63), potentially introducing attrition bias and limiting the ability to detect rare late complications.

## 5 Conclusion

Our findings provide clinically relevant evidence on procedure-related maternal outcomes following HAL when performed within an experienced multidisciplinary tertiary-center setting. Although PAS was associated with substantially greater surgical complexity, early procedure-related maternal morbidity remained comparable between the PAS and non-PAS groups, and PAS status was not independently associated with early non-transfusion major maternal morbidity after adjustment for baseline clinical characteristics. No severe ischemic complications were observed during hospitalization or reported by women who completed the structured long-term questionnaire.

## Data Availability

The datasets generated and/or analyzed during the current study are not publicly available due to patient confidentiality and applicable data protection requirements. De-identified data may be made available by the corresponding author upon reasonable request, subject to institutional approval and applicable ethical and legal requirements.
