# Supplementary material for: Maternal safety outcomes following bilateral hypogastric artery ligation in placenta accreta spectrum and severe obstetric hemorrhage: a retrospective tertiary-center cohort
Source: Front Med (Lausanne). 2026 Oct 21;13:1962336. doi: 10.3389/fmed.2026.1962336 (PMC13753764; doi:10.3389/fmed.2026.1962336)
Supplement: Supplementary file 1 [file Table1.docx]

This supplementary material provides a detailed technical description of bilateral hypogastric (internal iliac) artery ligation (HAL) as performed in our cohort. The technique uses a systematic transperitoneal retroperitoneal approach, with emphasis on anatomical identification and surgical maneuvers to minimize urological and major vascular injury.

Step 1: Palpation and identification of landmarks

The procedure commences with manual exploration of the pelvic sidewalls to identify the arterial pulse through the parietal peritoneum. The surgeon palpates the common iliac artery and follows its course distally to the bifurcation. The external iliac artery is identified as a longitudinal pulsating structure. In cases of significant pelvic edema or hypervascularity associated with PAS, the psoas muscle and the pelvic brim serve as the primary stable anatomical landmarks for orientation.

Step 2: Opening the parietal peritoneum

A longitudinal incision of the parietal peritoneum, approximately 4–6 cm in length, is made parallel to the iliac vessels. The peritoneum is elevated with vascular forceps to minimize the risk of injury to the underlying structures and provide access to the retroperitoneal space.

Step 3: Verification and development of the paravesical space

Definitive identification of the internal iliac artery, the external iliac artery, and the ureter is a critical safety requirement. This is achieved through the systematic development of the paravesical space, which allows for the safe medial retraction of the ureter and provides a clear view of the vascular bifurcation. The surgeon must visually confirm the divergent courses of the internal and external iliac arteries to avoid inadvertent ligation of the external iliac artery.

Step 4: Cleaning and mobilizing the artery

The internal iliac artery is carefully dissected approximately 2–3 cm distal to the bifurcation. Mobilization is achieved by blunt and sharp dissection within the paravesical and pararectal spaces to ensure adequate exposure. A retroarterial passage is then developed using a right-angled dissector, with careful separation of the vessel from the surrounding connective tissue to avoid injury to adjacent venous branches.

Step 5: Passing the suture and ligation

A right-angled dissector or Deschamps needle is used to pass the suture through the previously developed retroarterial plane. Particular care is taken during this maneuver to avoid injury to the underlying internal iliac vein. Double ligation is routinely performed using either heavy non-absorbable silk or synthetic absorbable suture material (e.g., polyglactin 0), with the ligatures secured by separate surgical knots.

Step 6: Final hemostatic check and safety verification

Following ligation, the operative field is inspected for hemostasis. Patency of the external iliac artery is confirmed by palpation distal to the ligation site, and the integrity and course of the ureter are re-verified. The retroperitoneal spaces are then inspected for residual bleeding before proceeding with subsequent surgical steps.
