# Supplementary material for: Maternal safety outcomes following bilateral hypogastric artery ligation in placenta accreta spectrum and severe obstetric hemorrhage: a retrospective tertiary-center cohort
Source: Front Med (Lausanne). 2026 Oct 21;13:1962336. doi: 10.3389/fmed.2026.1962336 (PMC13753764; doi:10.3389/fmed.2026.1962336)
Supplement: Supplementary file 2 [file Table2.docx]

Table S1. Baseline characteristics according to approach for long-term follow-up after HAL

| **Parameter** | **Women approached for long-term follow-up** | **Women not approached for long-term follow-up** | **p** |
| --- | --- | --- | --- |
|  | **N-75 (54.7%)** | **N-62 (45.3%)** |  |
| **Median mother age at birth (years)** | 34 (31 - 37) | 33 (31 - 36) | 0.170^*^ |
| **BMI (kg/m^2^)** | 25 (24 - 28) | 26 (24 - 27) | 0.954^*^ |
| **Gestation week at delivery** | 36 (33 - 39) | 36 (33 - 38) | 0.728^*^ |
| **PAS status** | | | |
| **Non-PAS group** | 48 (64.0%) | 44 (71.0%) | 0.466^+^ |
| **PAS group** | 27 (36.0%) | 18 (29.0%) |  |
| **Placenta praevia** | | | |
| No | 37 (49.3%) | 34 (54.8%) | 0.607^+^ |
| Yes | 38 (50.7%) | 28 (45.2%) |  |
| **History of previous uterine surgery** | | | |
| No | 38 (50.7%) | 34 (54.8%) | 0.731^+^ |
| Yes | 37 (49.3%) | 28 (45.2%) |  |
| **Cesarean section** | | | |
| No | 12 (16.0%) | 12 (19.4%) | 0.656^+^ |
| Yes | 63 (84.0%) | 50 (80.6%) |  |
| **Hysterectomy** | | | |
| No | 36 (48.0%) | 31 (50.0%) | 0.865^+^ |
| Yes | 39 (52.0%) | 31 (50.0%) |  |
| **HAL indication** | | | |
| Therapeutic | 41 (54.7%) | 38 (61.3%) | 0.489^+^ |
| Prophylactic | 34 (45.3%) | 24 (38.7%) |  |
| **Parity** | | | |
| Para 0 | 21 (28.0%) | 22 (35.5%) | 0.592^+^ |
| Para 1 | 37 (49.3%) | 29 (46.8%) |  |
| Para 2 or more | 17 (22.7%) | 11 (17.7%) |  |

Table S1 presents baseline characteristics by participation in research on long-term patient-reported outcomes (LTO) following HAL. Numerical variables are reported as median (lower and upper quartiles). Categorical variables are reported as frequency (n) and column (%). The bold-emphasized variables show statistically significant (at a 5% significance level) differences in proportion between groups.

Statistics: *Mann-Whitney U test, +Fisher's exact test
